# Feasibility of Markerless Motion Capture for Three-Dimensional Gait Assessment in Community Settings

**DOI:** 10.3389/fnhum.2022.867485

**Published:** 2022-06-09

**Authors:** Theresa E. McGuirk, Elliott S. Perry, Wandasun B. Sihanath, Sherveen Riazati, Carolynn Patten

**Affiliations:** ^1^Biomechanics, Rehabilitation, and Integrative Neuroscience Lab, Department of Physical Medicine and Rehabilitation, School of Medicine, University of California, Davis, Sacramento, CA, United States; ^2^UC Davis Healthy Aging in a Digital World Initiative, a UC Davis “Big Idea”, Sacramento, CA, United States; ^3^Center for Neuroengineering and Medicine, University of California, Davis, Davis, CA, United States; ^4^Veterans Affairs Northern California Health Care System, Martinez, CA, United States

**Keywords:** markerless motion capture, deep learning, gait analysis, kinematics, spatiotemporal parameters, neurorehabilitation, digital biomarkers, feasibility

## Abstract

Three-dimensional (3D) kinematic analysis of gait holds potential as a digital biomarker to identify neuropathologies, monitor disease progression, and provide a high-resolution outcome measure to monitor neurorehabilitation efficacy by characterizing the mechanisms underlying gait impairments. There is a need for 3D motion capture technologies accessible to community, clinical, and rehabilitation settings. Image-based markerless motion capture (MLMC) using neural network-based deep learning algorithms shows promise as an accessible technology in these settings. In this study, we assessed the feasibility of implementing 3D MLMC technology outside the traditional laboratory environment to evaluate its potential as a tool for outcomes assessment in neurorehabilitation. A sample population of 166 individuals aged 9–87 years (mean 43.7, S.D. 20.4) of varied health history were evaluated at six different locations in the community over a 3-month period. Participants walked overground at self-selected (SS) and fastest comfortable (FC) speeds. Feasibility measures considered the expansion, implementation, and practicality of this MLMC system. A subset of the sample population (46 individuals) walked over a pressure-sensitive walkway (PSW) concurrently with MLMC to assess agreement of the spatiotemporal gait parameters measured between the two systems. Twelve spatiotemporal parameters were compared using mean differences, Bland-Altman analysis, and intraclass correlation coefficients for agreement (ICC_2,1_) and consistency (ICC_3,1_). All measures showed good to excellent agreement between MLMC and the PSW system with cadence, speed, step length, step time, stride length, and stride time showing strong similarity. Furthermore, this information can inform the development of rehabilitation strategies targeting gait dysfunction. These first experiments provide evidence for feasibility of using MLMC in community and clinical practice environments to acquire robust 3D kinematic data from a diverse population. This foundational work enables future investigation with MLMC especially its use as a digital biomarker of disease progression and rehabilitation outcome.

## Introduction

Access to accurate methods for human motion capture in clinical environments is motivated by the need to understand normal and pathological movement (Andriacchi and Alexander, 2000). Tools available in the clinic, such as pressure-sensitive mats, are well validated (Bilney et al., 2003; Menz et al., 2004; Webster et al., 2005), but limited to measurement of spatiotemporal parameters of gait. Wearable sensors, such as inertial measurement units (IMU), can be used to track gait characteristics including joint angles (Rapp et al., 2021), however the joint angle computation is challenging, and the sensors are affected by environmental features such as magnetic fields. The three-dimensional (3D) quantification of human gait informs rehabilitation and the progression of disease by characterizing the mechanisms underlying gait impairments (Ferrarin et al., 2005; Mundermann et al., 2005; Jonkers et al., 2009; Albani et al., 2014; Pistacchi et al., 2017).

Marker-based motion capture (MBMC) laboratories that conduct 3D gait analysis provide carefully validated measures, although some concessions are made. Study participants are required to travel to the lab, commit time, wear specialized clothing and shoes, and be extensively instrumented with reflective markers. It is often necessary to adjust or minimize clothing to expose anatomical landmarks, which can limit the pool of individuals willing to participate. Subjects must then attempt to walk “normally” under observation in an unfamiliar setting. As a result, there has long been a question regarding the ecological validity of gait data collected via motion capture in these dedicated laboratories (Hutchinson et al., 2019; Mc Ardle et al., 2021). Measurement of joint kinematics in a representative sample population is desirable yet to date has remained elusive. Additionally, racial and ethnic minorities have historically been underrepresented in clinical research (McCarthy, 1994). Work is being done to provide a more robust and complete understanding of the potential gait differences among diverse individuals in the population (Blanco et al., 2012). Taken together, gait assessment tools enabling high resolution measurement in participant-facing environments offer an opportunity to study a more representative sample population by reducing some of these economic, social, and cultural barriers. Furthermore, bringing 3D gait analysis directly into the clinical environment will increase its use as a clinical outcome measure and digital biomarker of pathology and disease progression. There is a need for 3D motion capture technology that can be used outside the controlled motion capture laboratory environment.

Over the last three decades, the fields of computer vision and machine learning have developed marker-free techniques to identify and track human movement (Hogg, 1983; Corazza et al., 2006; Martinez et al., 2018). Neural network-based methods for pose estimation have made a significant impact on the field making it possible to define and track anatomical landmarks without the use of markers (Mathis et al., 2018; Cronin, 2021). Research has quickly started to examine open-source frameworks such as OpenPose and DeepLabCut using one or two camera array systems to measure spatiotemporal gait parameters (Stenum et al., 2021; Lonini et al., 2022). One study utilizing a Kinect V2 found the technology performed reasonably well as a tool for measuring spatiotemporal gait parameters, but not joint kinematics (Mentiplay et al., 2015).

In the current study we implemented Theia3D (Theia Markerless Inc., Kingston, ON, Canada), which uses deep convolutional neural networks to perform 3D human pose estimation. This technology has been developed, tested for reliability, and validated with respect to traditional MBMC methods in a controlled laboratory environment by investigating a small sample of healthy, young adults. In the developers’ hands, the Theia3D MLMC system produced reliable 3D gait kinematics with lower inter-session variability than those reported by MBMC methods (Kanko et al., 2021a). Furthermore, Theia3D demonstrated good to excellent agreement for spatiotemporal parameters when compared to parameters produced by both a MBMC system and a pressure-sensitive gait mat (Kanko et al., 2021b). Lower limb 3D kinematic data extracted from concurrent MBMC and Theia3D MLMC systems were compared, reporting an average root mean square difference less than 2.5 cm for all corresponding joint centers except hip which was 3.6 cm (Kanko et al., 2021c). Here we expand on these findings by investigating the use of Theia3D to quantify 3D gait kinematics outside the controlled laboratory environment on a moderately large sample population of varied age and health history.

The aim of this study was to assess the feasibility of implementing 3D MLMC technology in community accessible environments (i.e., community centers, clinic-adjacent facilities, parks, etc.). Our goal was to successfully mobilize 3D MLMC technology to a variety of locations and perform overground gait experiments on a diverse sample population. In addition, we evaluated agreement of spatiotemporal gait parameters concurrently measured by MLMC and pressure-sensitive walkway systems.

## Materials and Methods

### Participants

We studied 166 participants over a 3-month period at six distinct locations in the community. All individuals studied met the following inclusion criteria: ability to provide informed consent, follow three-step commands, and ambulate 15 m independently, even if requiring an assistive device or brace. Participants provided electronic written informed consent prior to enrollment and participation, with minors providing assent in addition to the consent of a parent/legal guardian. All procedures were approved by University of California, Davis Institutional Review Board (#1386142) and conducted according to the Declaration of Helsinki.

As participants were recruited directly from their daily activities in the community, they wore their own clothing and footwear. Individuals wearing long-hemmed skirts (i.e., mid-calf or longer) were excluded as the joints at the knee and hip could not be tracked by the deep learning algorithm. In accordance with COVID-19 precautions, all participants wore face masks covering the nose and mouth.

### Experimental Setup and Procedure

**FIGURE 1:**
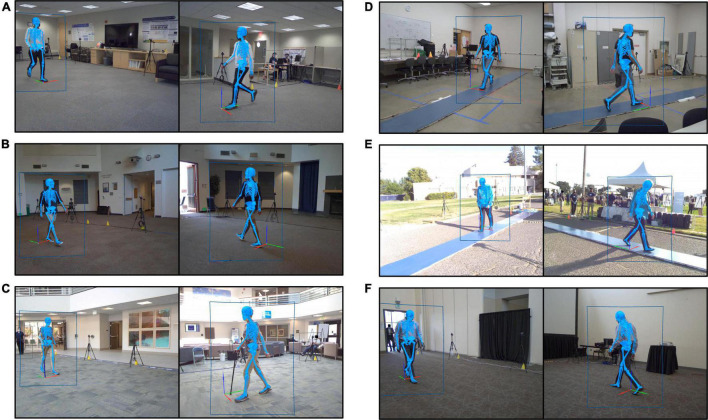
Still images recorded by the markerless motion capture video cameras during gait experiments performed at each of the six experimental locations. Each image shows a participant walking. Human pose identification is indicated by the blue rectangle outlining the participant. The estimated three-dimensional pose generated by Theia3D is represented by the blue skeleton overlaid on the subject image. **(A)** Location 1, spare office-style room. **(B)** Location 2, community event room. **(C)** Location 3, clinic-adjacent lobby. **(D)** Location 4, spare storage-style room. **(E)** Location 5, outdoor event at a sports field. **(F)** Location 6, conference center breakout room.

Six locations (Figure 1) were scouted and identified for experiments. The ideal space offered: (1) a flat surface with minimum dimensions 9 by 7.5 m, (2) sufficient illumination, (3) access to electrical power, and (4) visible to community foot traffic. The first three criteria ensured the experiment was successfully instrumented, while the last increased the likelihood of participant recruitment. Minimum space dimensions were determined by the camera locations required to create a capture volume for overground walking. Over a 3-month period, equipment was transported between these six locations based on space availability, with some sites visited multiple times. Locations open to public foot traffic required equipment be broken down and securely stored at the end of each experimental session. Care was taken to control public foot traffic near the camera tripods.

The markerless motion capture (MLMC) system was comprised of eight video cameras (Basler ace acA1300-75gc GigE, Ahrensburg, Germany) positioned on tripods placed along the perimeter of a capture volume approximately 6 m long, 4 m wide, and 2 m high. Cameras were positioned in an elliptical formation, with four cameras along each side of the walking path; cameras nearest the ends of the walking path were shifted 0.5 m to the center. Orange sports cones were placed 10 m apart to mark the targeted start and end of the walking path. An intrinsic camera calibration was performed once, prior to all experiments, using a black-and-white checkerboard pattern (10 by 6 grid of 0.077 m squares). Extrinsic camera calibrations were performed at the beginning of each experimental session following camera positioning, lens focus, and aperture checks. An extrinsic cube calibration object (0.655 m) was centrally positioned in the capture volume with the +Y/–Y global reference directed in line with the walking path and +Z directed toward the sky. Video data from the cameras were synchronized and recorded at 60 Hz using AccuPower 4.0 software (v1.3.6.1978 Treadmetrix, Park City, UT, United States). The resulting two-dimensional (2D) video data were calibrated using Theia3D (v2021.2.0.1675).

In a corollary experiment, we assessed agreement of spatiotemporal parameters measured by the MLMC system and a pressure-sensitive walkway (PSW) (Stepscan, Charlottetown, PE, Canada). We used an 8.4 m long PSW positioned on the floor central to the motion capture volume at two of the six sites (Locations 4 and 5). The PSW was constructed using 14 tiles (0.6 m square); the center of the walkway was instrumented (6 tiles, each with 14,400 pressure sensors), while the remaining were dummy tiles – identical in appearance but not instrumented. Data from the PSW system were sampled at 100 Hz.

Participant details including height, weight, and leg-length were measured onsite using a portable stadiometer (213 Seca, Chino, CA, United States), digital scale (PD759L, MyWeight, Phoenix, AZ, United States), and measuring tape (length 150 cm). Demographics and health history were gathered using an electronic questionnaire. Investigators defined the categories of the demographics for ethnicity, race, biological sex, and gender. These data were managed using Research Electronic Data Capture (REDCap), an electronic data capture tool hosted at the University of California, Davis Clinical and Translational Sciences Center (Harris et al., 2009, 2019).

The motion capture experiment involved two overground walking tasks: self-selected (SS) and fastest comfortable (FC) walking. For both tasks, the participant was positioned at one end of the walking path and instructed to walk across the room toward the orange cone positioned at the opposite end of the walking path. The experimenter provided a verbal “Go” command to signal the participant to start each walking pass. Participants were instructed to, “walk as you usually do” for the SS walking task and to “walk as fast as possible, as if you are in a crosswalk and notice oncoming traffic” for the FC walking task. To acquire a minimum of 12 full gait cycles per leg for each task, participants repeated approximately 6–8 passes per task with the SS task completed first. At locations where both the MLMC and PSW systems were implemented, kinematic and spatiotemporal data were recorded concurrently.

### Data Analysis

Feasibility measures for this experiment were developed based on guidelines outlined by Bowen et al. (2009), with three areas of focus: (1) the *implementation* of MLMC in community spaces offering the research team limited environmental control, (2) the *practicality* of mobilizing MLMC with respect to investment from both the research team and study participants, and (3) the *expansion* of MLMC from lab to public living domain, including testing a diverse population. An overview of these measures is provided in Table 1. Feasibility measures were structured as yes or no questions. The success of each feasibility measure was considered separately for each experimental site (Locations 1–6).

**TABLE 1:** Overview of feasibility measures assessed at each of the six experimental locations.

Feasibility measures	Questions	Locations						Success rate	
		1	2	3	4	5	6		
		
Implementation								29/30	96.7%
Location features	Does the location contain enough space to produce the designed motion capture volume? (min 9 × 7.5 m for this OG experiment)	Yes	Yes	Yes	Yes	Yes	Yes	6/6	100%
	Does the space have an accessible power source to operate equipment within reach of 20-foot extension cord?	Yes	Yes	Yes	Yes	Yes	Yes	6/6	100%
	Does the space have a constant, sufficient light source?	Yes	No	Yes	Yes	Yes	Yes	5/6	83.3%
System mobility	Could equipment and supplies be packed, transported, and couriered to and from testing sites?	Yes	Yes	Yes	Yes	Yes	Yes	6/6	100%
Data management	Could recorded data be securely stored?	Yes	Yes	Yes	Yes	Yes	Yes	6/6	100%

**Practicality**								**38/42**	**90.5%**

Participant investment	Could the participant complete the experiment without additional travel?	Yes	Yes	Yes	Yes	Yes	Yes	6/6	100%
	Was the average time commitment under 30 min? (This total time includes wait time, IRB consent, electronic questionnaire, and gait experiment)	Yes	Yes	Yes	Yes	No	Yes	5/6	83.3%
	Were participants tested in the clothes in which they arrived? (With the exception of long skirts.)	Yes	Yes	Yes	Yes	Yes	Yes	6/6	100%
Research investment	Could the system be unpacked, set up, calibrated and ready for use within 1 hour of arriving on site?	Yes	Yes	Yes	Yes	Yes	Yes	6/6	100%
	Could the system be mobilized, set up and experiment performed with a minimum of four people?	Yes	Yes	Yes	Yes	No	Yes	5/6	83.3%
	Was it possible to access and recruit a minimum of 10 participants each time the equipment was set up?	Yes	Yes	Yes	Yes	Yes	No	5/6	83.3%
	Could the 3D kinematic data be quickly extracted and efficiently processed?	Yes	No	Yes	Yes	Yes	Yes	5/6	83.3%

**Expansion**								**24/24**	**100%**

3D Kinematics	Did the MLMC system produce a full-body 3D kinematic model outside a controlled laboratory setting? (min 3 consecutive gait cycles of full body kinematics for each pass through the capture volume)	Yes	Yes	Yes	Yes	Yes	Yes	6/6	100%
	Did the MLMC system produce a full-body 3D kinematic model in the presence of background activity?	Yes	Yes	Yes	Yes	Yes	Yes	6/6	100%
Diverse population	Did location provide access to a sample population representative of the surrounding metropolitan area?	Yes	Yes	Yes	Yes	Yes	Yes	6/6	100%
	Did the MLMC system produce a full-body 3D kinematic model for participants using assistive device (walker, cane)?	Yes	Yes	Yes	Yes	Yes	Yes	6/6	100%

To extract 3D kinematic data from the MLMC system, synchronized videos of identical length were processed en masse using the Theia3D companion application TMBatch. A model with 6 degrees-of-freedom (DOF) at the pelvis, 3 DOF at the hip, 3 DOF at the knee, and 3 DOF at the ankle was used to estimate a 3D pose of the subject completing the walking task (Figure 1). The 4 × 4 pose estimates for each body segment were exported to Visual3D Professional (v2021.06.02, C-Motion, Inc., Germantown, MD, United States) for further analysis. The skeletal model automatically generated in Visual3D was edited to add a position reference for the alternating +Y and –Y walking directions. Theia3D can detect and generate multiple 3D poses when additional persons are visible on camera. To identify the study participant’s estimated 3D pose from this group, an automated algorithm was developed and run in Visual3D which referenced 3D pose position relative to the global origin. The selected model was used to calculate joint center positions which represent direct estimates of those positions from the deep learning algorithm. Lower limb joint angles were calculated using a Cardan sequence equivalent to the joint coordinate system (Grood and Suntay, 1983). Gait events [i.e., initial contact (IC) and toe off (TO)] were identified using a coordinate-based algorithm measuring the maximal displacement of the heel and toe from the pelvis in the direction of walking progression (Zeni et al., 2008) with quality checks of event detection performed in Visual3D. Time-series segment positions, kinematic measurements, and gait events were exported to MATLAB (v2020a, Mathworks, Natick, MA, United States) for further analysis.

Spatiotemporal gait parameters were measured parallel to the direction of walking progression (Huxham et al., 2006). Metrics included: cadence, speed, step length, stride length, stride width, step time, stride time, stance time, swing time, single limb support time, and double limb support time. MLMC system values were defined in MATLAB using the foot segment position and gait events extracted from Visual3D. PSW system values were automatically defined in the Stepscan software (v2.5.26.0, Stepscan Technologies, Inc., Charlottetown, PE, Canada) using the position and timing of footprints and exported to MATLAB for further analysis. Data quality checks were performed in the Stepscan software to exclude footprints which did not occur entirely within the pressure-sensitive walking area. Stepscan provides both the contralateral and ipsilateral step associated with each full stride. Only the ipsilateral step associated with each stride was included in this analysis.

To determine the likely magnitude of the difference in spatiotemporal parameters produced by the MLMC and PSW systems, the mean difference and percent error of absolute difference between the two systems were calculated. Mean differences are defined relative to the PSW system where a negative value indicates the PSW value is less than the MLMC value. The spread of mean differences with 95% limits of agreement (LOA) was assessed using the Bland-Altman method (Bland and Altman, 1986). Intraclass correlation coefficients (ICC) for agreement (2,1) and consistency (3,1) were calculated in Statistical Product and Service Solutions (SPSS) (v27, IBM SPSS Statistics, Armonk, NY, United States) to determine inter-instrument reliability for each pair of spatiotemporal metrics (Shrout and Fleiss, 1979; McGraw and Wong, 1996). ICC values < 0.5 were interpreted as poor, 0.5–0.75 as moderate, 0.75–0.9 as good, and >0.9 as excellent (Portney and Watkins, 2009).

## Results

### Feasibility Measures

In the area of implementation, five feasibility measures were considered at six experimental locations resulting in an overall success rate of 96.7% (29/30). All six locations afforded the required physical location features except Location 2 which did not provide sufficient illumination. Both mobility of the MLMC system and data management were implemented with a 100% success rate. In the area of practicality, an overall success rate of 90.5% (38/42) was demonstrated. Considerations for the minimization of participant investment was fully successful at all experimental locations except Location 5. The investment of the research team was 100% successful with respect to a fast and efficient setup time. Consideration of staff number to participant yield ratio was 83.3% successful. Location 5 required an expanded research team (12 staff), and Location 6 only involved three participant experiments. Procedures to process data quickly and efficiently were 83.3% successful. Data collected at Location 2 required additional attention during analysis. Lastly, in the area of expansion, an overall success rate of 100% (24/24) was observed. Full-body 3D kinematics were produced at all experimental locations regardless of background activity or the presence of assistive devices including canes and walkers. A diverse population, representative of the surrounding metropolitan region, was successfully enrolled and tested at all six locations. An overview of the feasibility results can be found in Table 1.

### Pressure-Sensitive Walkway Comparison

A subset of 46 participants (32 female) aged between 13 and 86 years [mean 45.9, (S.D. 22.9)] was used to compare spatiotemporal parameters measured concurrently with both the MLMC and PSW systems. The average number of gait cycles identified by the PSW system was 17.7 ± 5.4 for SS and 16.7 ± 5.5 for FC as compared to 25.1 ± 5.4 for SS and 25.8 ± 6.95 for FC identified by the MLMC system.

Overall, mean spatiotemporal values reported by both systems were quite similar. Tables 2A,B report the means and comparative difference measures between the two systems for the SS and FC tasks, respectively. The majority of these parameters measured small differences between the PSW and MLMC systems although values for cadence, speed, step time, stride time, and stride length appeared practically identical (mean absolute difference < 1% of mean PSW values). Cadence revealed mean differences < 0.29 steps/min for both SS and FC, and Bland–Altmann LOA approximately ± 2 steps/min. Speed revealed a mean difference of –0.01 m/s for SS, essentially 0.00 m/s for FC, and an approximate LOA of ±0.03 m/s. Step length and stride length both revealed mean differences of –0.6 cm for SS and –0.2 cm for FC, respectively, and approximate LOA of ±2.0 cm. Stride width revealed a mean difference of –1.2 cm for SS and –1.8 cm for FC respectively. The LOA ranged –5.2 – 2.9 cm for SS and –5.3 – 2.2 cm for FC. All temporal measures revealed mean differences of 0.02 s or less. Step time revealed mean differences of –0.001 s and LOA of ±0.01 s for both tasks. Stride time revealed a mean difference of 0.001 s and LOA of ±0.02 s for SS and –0.002 s and LOA of ±0.01 s for FC. Stance time produced a mean difference of –0.02 s and swing time a mean difference of 0.02 s for both tasks. The LOA for stance time ranged between –0.05 – 0.02 s while the LOA for swing time ranged from –0.02 – 0.05 s. Both 1^st^ double limb support time and 2^nd^ double limb support time revealed mean differences of –0.02 s and an LOA range approximately –0.05 – 0.02 s for both walking tasks. Single limb support time and swing time, which capture the same epoch characteristics in opposite legs, performed similarly. Both single limb support time and swing time revealed mean differences of 0.02 s and an LOA range approximately –0.02 – 0.05 s.

**TABLE 2A:** Mean, standard deviation (SD), mean difference and standard deviation of differences, Bland–Altman limits of agreement (LOA) over a 95% confidence interval (CI), intraclass correlation coefficient (ICC) values, lower bounds and upper bounds of agreement [ICC_(2,1)_] and consistency [ICC_(3,1)_] for the comparison of spatiotemporal measures extracted by the pressure-sensitive walkway (PSW) and markerless motion capture (MLMC) for the self-selected (SS) walking speed task.

	PSW Mean (SD)	MLMC Mean (SD)	Mean (SD) Diff, % error	LOA, 95% CI		Agreement: ICC_(2,1)_ (95% CI)	Consistency: ICC_(3,1)_ (95% CI)
				Lower	Upper		
Cadence (steps/min)	109.72	109.43	0.29, 0.75%	–0.02	0.6	0.997 (0.994–0.998)	0.997 (0.995–0.998)
	(9.77)	(9.65)	(1.04)				
Speed (m/s)	1.23	1.23	–0.01, 0.95%	–0.01	–0.002	0.999 (0.998–0.999)	0.999 (0.998–1)
	(0.23)	(0.23)	(0.01)				
Step Length (m)	0.670	0.676	–0.006, 1.25%	–0.008	–0.004	0.998 (0.988–0.999)	0.999 (0.998–0.999)
	(0.106)	(0.105)	(0.007)				
Stride Length (m)	1.343	1.349	–0.006, 0.76%	–0.009	–0.003	0.999 (0.998–1)	0.999 (0.999–1)
	(0.214)	(0.211)	(0.011)				
Stride Width (m)	0.125	0.136	–0.012, 17.14%	–0.018	–0.005	0.874 (0.700–0.939)	0.899 (0.818–0.944)
	(0.037)	(0.031)	(0.021)				
Step Time (s)	0.55	0.55	–0.001, 0.76%	–0.003	0.001	0.997 (0.995–0.998)	0.997 (0.995–0.998)
	(0.05)	(0.05)	(0.006)				
Stride Time (s)	1.11	1.11	0.001, 0.5%	–0.001	0.004	0.998 (0.997–0.999)	0.998 (0.997–0.999)
	(0.11)	(0.10)	(0.01)				
Stance Time (s)	0.72	0.73	–0.02, 2.84%	–0.02	–0.01	0.976 (0.875–0.991)	0.986 (0.974–0.992)
	(0.08)	(0.08)	(0.02)				
1^st^ Dbl. Sup. Time (s)	0.16	0.18	–0.02, 13.16%	–0.02	–0.01	0.803 (0.397–0.916)	0.866 (0.757–0.926)
	(0.03)	(0.03)	(0.02)				
Single Sup. Time (s)	0.39	0.37	0.02, 5.27%	0.01	0.02	0.841 (0.365–0.939)	0.903 (0.825–0.946)
	(0.04)	(0.03)	(0.02)				
2^nd^ Dbl. Sup. Time (s)	0.16	0.18	–0.02, 13.74%	–0.02	–0.01	0.813 (0.169–0.932)	0.897 (0.814–0.943)
	(0.03)	(0.03)	(0.02)				
Swing Time (s)	0.39	0.37	0.02, 5.15%	0.01	0.02	0.859 (0.342–0.949)	0.920 (0.856–0.956)
	(0.04)	(0.03)	(0.02)				

**TABLE 2B:** Mean, standard deviation (SD), mean difference and standard deviation of differences, Bland–Altman limits of agreement (LOA) over a 95% confidence interval (CI), intraclass correlation coefficient (ICC) values, lower bounds and upper bounds of agreement [ICC_(2,1)_] and consistency [ICC_(3,1)_] for the comparison of spatiotemporal measures extracted by the pressure-sensitive walkway (PSW) and markerless motion capture (MLMC) for the fastest comfortable (FC) walking speed task.

	PSW Mean (SD)	MLMC Mean (SD)	Mean (SD) Diff, % error	LOA, 95% CI		Agreement: ICC_(2,1)_ (95% CI)	Consistency: ICC_(3,1)_ (95% CI)
				Lower	Upper		
Cadence (steps/min)	136.17	135.95	0.22, 0.84%	–2.53	2.97	0.997 (0.995–0.999)	0.997 (0.995–0.999)
	(13.86)	(13.93)	(1.40)				
Speed (m/s)	1.85	1.85	0.0006, 0.67%	–0.03	0.03	0.999 (0.999–1)	0.999 (0.999–1)
	(0.36)	(0.36)	(0.02)				
Step Length (m)	0.812	0.814	−0.002, 0.92%	–0.022	0.017	0.998 (0.997–0.999)	0.998 (0.997–0.999)
	(0.140)	(0.140)	(0.012)				
Stride Length (m)	1.624	1.637	−0.002, 0.61%	–0.029	0.023	0.999 (0.998–1)	0.999 (0.998–1)
	(0.280)	(0.281)	(0.017)				
Stride Width (m)	0.126	0.142	-0.018, 17.96%	–0.053	0.022	0.864 (0.52–0.945)	0.911 (0.836–0.951)
	(0.036)	(0.033)	(0.023)				
Step Time (s)	0.45	0.45	−0.0007, 0.83%	–0.01	0.01	0.997 (0.995–0.999)	0.997 (0.995–0.999)
	(0.04)	(0.05)	(0.005)				
Stride Time (s)	0.89	0.89	−0.002, 0.5%	–0.01	0.01	0.999 (0.998–0.999)	0.999 (0.998–1)
	(0.09)	(0.09)	(0.01)				
Stance Time (s)	0.56	0.57	−0.02, 3.6%	–0.05	0.02	0.972 (0.758–0.991)	0.986 (0.974–0.992)
	(0.07)	(0.06)	(0.02)				
1^st^ Dbl. Sup. Time (s)	0.11 (0.03)	0.13 (0.02)	−0.02, 20.23% (0.02)	–0.05	0.02	0.793 (0.294–0.918)	0.866 (0.744–0.93)
Single Sup. Time (s)	0.33	0.31	0.02, 6.34%	–0.02	0.05	0.8 (0.313–0.921)	0.871 (0.753–0.932)
	(0.03)	(0.03)	(0.02)				
2^nd^ Dbl. Sup. Time (s)	0.11 (0.03)	0.13 (0.02)	−0.02, 18.02% (0.02)	–0.05	0.02	0.821 (0.213–0.935)	0.899 (0.816–0.945)
Swing Time (s)	0.33	0.32	0.02, 5.56%	–0.02	0.05	0.857 (0.354–0.948)	0.918 (0.849–0.955)
	(0.03)	(0.03)	(0.02)				

Intraclass correlation coefficients for agreement [ICC_(2,1)_] indicated excellent agreement [ICC_(2,1)_ > 0.9] for cadence, speed, step length, stride length, step time, stride time, and stance time, and good agreement [ICC_(2,1)_ > 0.75] for stride width, 1^st^ double limb support time, 2^nd^ double limb support time, single limb support time, and swing time. The ICC_(2,1)_ lower bound of 0.169 for 2^nd^ double limb support time of the SS task indicates the possibility of poor agreement. Intraclass correlation coefficients for consistency [ICC_(3,1)_] indicated excellent consistency [ICC_(3,1)_ > 0.9] between instruments for cadence, speed, step length, stride length, step time, stride time, stance time, swing time and good agreement [ICC_(3,1)_ > 0.75] for stride width, 1^st^ double limb support time, 2^nd^ double limb support time, and single limb support time. Both stride width and single limb support time showed higher consistency in the FC task compared to the SS task.

## Discussion

### Considerations of Feasibility

#### Implementation

The successful implementation of 3D MLMC outside the lab was first and foremost based on the physical features of the space: floor dimensions, light source, and power access.

The minimum floor dimensions identified for this experiment (9 by 7.5 m) were informed by the requisite motion capture volume for our objectives. Methods to design the capture volume for a 3D MLMC system are similar to a traditional 3D MBMC lab where both the movement task (overground walking) and video camera specifications (8 cameras, 1280 by 1024 px resolution, 6 mm lens focal length) are considered (Winter, 2009). Additionally, in accordance with Theia3D specifications, the algorithm performs optimally when the height of the subject is at least 500 px. Considering these three features, our team optimized camera orientation to extend the motion capture volume, maximizing the number of gait cycles recorded in both directions with each pass. We operationalized our procedures to have participants start each walking pass outside the capture volume, ensuring they were up to speed by the time the biomechanical model was fully identified inside the capture volume. This approach enabled us to use data from every stride within the targeted walkway of the capture volume. The tradeoff with this design is a period of 1–2 m at both ends of the 10-m walkway where the model is not yet fully identified. While these attributes were the appropriate methodology for our purposes, it did introduce a need for additional quality checks during gait detection. Experiment configurations where the model remains within the capture volume (i.e., walking on a treadmill, isolated joint movements, or sit-to-stand) would likely not experience this issue.

To operate the MLMC equipment, access to a power source was a requirement we considered prior to selecting experimental locations. The power source needed to be accessible using a 20-foot extension cord. This necessity made the proximity of the power source a defining feature when planning the orientation of the experimental walkway within each unique space. To successfully perform experiments at Location 5, an outdoor space without access to a permanent power source, an electric generator was provided by the event coordinators.

Sufficient illumination of the environment proved vital to successful data collections. Even though it was not necessary to control the color of the subject’s clothing relative to background, we found it was necessary for the space to be well lit. Location 2 was a spacious community room that relied mostly on natural light that streamed through high windows and skylights. While it was possible to produce a 3D biomechanical model for all tested participants at this site, the model was consistent over a shorter capture volume as compared to the other five locations. As a result, each walking pass yielded fewer gait cycles at this site. However, Location 2 was adjacent to an arboretum with walking trails and public parking, making it an excellent source for recruiting participants into the study on location. For this reason, it might be worth returning to Location 2 with additional, portable light sources to determine how much experimental conditions could be improved. Location 5 also used sunlight for illumination. In this case, because the experiment was initiated in the late afternoon, and finished near dusk, data collected toward the end of the day required more hands-on attention to process.

The MLMC system can be mobilized. The ability for the research team to easily mobilize and transport equipment between sites also factored into the practicality of using the MLMC system outside the lab. All equipment and supporting supplies were efficiently packed and transported to all six locations using a single vehicle. When scouting locations for these experiments, ease of access was also considered in the form of ramps, elevators, and proximity from vehicle to the experimental setup.

Related to mobilization, the management of data file size and security are also a consideration for successful implementation. Depending on video sampling frequency and trial length, the raw video files, in combination with the processed data files created by Theia3D, can be much larger compared to marker-based data files. By design of the deep learning algorithm requirements, these video files include identifiable participant features, most notably facial features. As a result, both our institutional information security and privacy practices, and our IRB, required use of approved, secure data storage that can manage large files and be implemented in the field.

#### Practicality

Resolving that it is indeed possible to mobilize and implement MLMC outside the lab, we next consider whether such an application is practical for both the participant and the research team.

The time commitment required by participants was generally minimized with respect to traditional MBMC experiment methods. Participants were not required to travel to a dedicated experiment location as they were recruited on site. On average, participants spent 20 min with the research team during which they: provided informed consent, completed their health history/demographics questionnaire, and performed the walking experiment. Location 5 was an exception. This location involved a university sponsored event limited to a 2 hour period, attended by numerous individuals interested in and willing to participate, resulting in a long queue. In parallel to the data collection, the research team also processed data on site to provide each participant with an individualized gait report. At this event, the research team achieved a maximum of 8.5 complete gait studies per hour! In comparison, this threshold is unlikely to be achieved by MBMC without additional resources in the form of multiple reflective marker sets, research personnel trained to place markers, participants willing to commit to a longer preparation time, and the adjustment/removal of clothing to track markers. In fact, experiments performed at Location 5 highlight the potential of MLMC to efficiently capture a large quantity of full-body 3D kinematic gait (motion) studies.

Participants were enrolled onsite and in the moment, thus were studied wearing their normal attire, which ranged from business casual trousers and shoes to hiking shorts. Monochromatic clothing did not appear to challenge the processing algorithm. However, long-hemmed skirts did not allow specific features of the legs to be tracked. When recruiting individuals in the community, those wearing long-hemmed skirts (i.e., mid-calf or longer) were given the option to change into shorts in order to participate in the study. We recognize that use and, perhaps more importantly, type of footwear affect a person’s gait (Franklin et al., 2015; Wiedemeijer and Otten, 2018). However, the focus of this study was to measure gait “in the wild” with as little adjustment and interaction from the research team as possible. People wearing heeled shoes were prompted to remove their shoes and walk barefoot. However, if they opted to walk in heeled shoes, the data were still collected but tagged for special consideration. Anecdotally, we noted that a 3D biomechanical model did appear to appropriately estimate the ankle and foot segment of participants wearing high-heeled shoes. This feature bears further investigation in the future.

The entirety of this study was conducted in public spaces at the height of a contagious global pandemic. Without the necessity to instrument the body with markers or IMU’s, we had limited need to come in close physical contact with participants enabling us to continue our active research program despite pandemic restrictions. The deep learning algorithm developed by Theia3D was trained to identify 51 salient features consisting of joint locations and other identifiable surface features of the human body including the face. Prior to the start of this study, we investigated how the algorithm would respond if the mouth and nose were occluded by a face mask. While these results are not presented here, this preparatory exploration did inform our methods. As such, all subjects wore face masks over mouth and nose while completing walking tasks, and the deep learning algorithm successfully resolved facial features to enable biomechanical models.

Mobilizing the MLMC system does increase the burden to the research team in terms of resources to set up and break-down equipment for each experimental session. However, once onsite at all six locations, the system could be unpacked, set-up, calibrated, and ready for use in under 1 hour. This short setup time left a full 8-h day open to collect data. On average, we successfully recruited and studied 15.6 individuals per day on location. We typically went into the field with four lab members. The unique circumstances of Location 5 (high-density crowd for a time-limited period) led the team to expand to a staff of twelve. Location 6 was also a unique setup for the opposite reason. Here, the MLMC system was setup in an auxiliary space to our lab with the express purpose of only testing a small handful of individuals, thus the minimum threshold of ten participants was not met. Testing in locations with high foot traffic, such as Locations 2 and 5, show the quantity and diverse quality of the population which can be accessed each day, easily justifying the researcher’s investment to mobilize the MLMC system.

We utilized batch processing methods to collect, analyze, report, and securely back-up the data collected onsite at each experimental location quickly and efficiently. Tools including unique MATLAB programs and the Microsoft Windows command Robust File Copy (robocopy) were used to transfer files between the AccuPower, Theia3D, and Visual3D file workspaces prior to running respective pipelines on the data in those spaces. When used all together, it was possible for us to quickly process data for two applications: (1) collect a full day’s worth of data, then batch process it after returning to our home workspace, and (2) process data for each individual as it was collected. To reduce the waiting time for interested individuals, we employed the first method at most of our experimental locations, especially where foot-traffic was high. The second method was employed at Location 6 where we demonstrated it was possible to collect, process, analyze, and report measures of gait in under an hour. The insufficient illumination at Location 2 also appeared to affect the performance of the Theia3D algorithm. Data collected at Location 2 could not be batch processed because they required considerable hands-on processing attention which extended the overall processing time and practicality consideration. The ability to process data and produce results quickly and efficiently demonstrates the practical use of this system to researchers with studies collecting large quantities of 3D kinematic data in the field. This capacity is also of interest for clinicians who require fast turnaround of 3D kinematic gait measures to aid in neurorehabilitation assessments.

#### Expansion

The MLMC system demonstrated successful expansion outside the controlled environment of a typical motion capture laboratory. Specific to this overground walking experiment, we established a minimum requirement of three consecutive gait cycles with full-body 3D kinematics to be recorded for every participant at all six locations. On average, each pass through the capture volume recorded 5 and 4 gait cycles for the SS and FC walking tasks, respectively. The number of gait cycles acquired with each pass was dependent on the individual’s leg length and gait speed. At five of the six locations, it was possible to implement the camera setup described in this paper and successfully create a motion capture volume with a 6-m-long walkway. Even though Location 2 provided the necessary floor dimensions to recreate the camera setup, insufficient illumination resulted in a shorter walkway (approximately 4 m) where the full-body 3D kinematic model was cleanly produced. This led to fewer full gait cycles detected with each pass, yet still met the minimum requirement of three consecutive gait cycles.

Background activity is a necessary consideration of the feasibility of using the MLMC system in community and clinical environments where extraneous activities are constant throughout the day. The Theia3D algorithm produced a full-body 3D kinematic model for all participants, regardless of background activity, both inside and outside the motion capture volume. Background activity unassociated with our experiments (i.e., foot traffic, people seated in waiting rooms, regular workplace movements) was captured during data collections at all six experimental locations. For example, at Location 2, individuals were continuously moving along the perimeter of the room, outside the camera capture space. We staged a subject intake station (measurement of height, weight, and leg length) along one side of the room, and a seated waiting area for companions of study participants on the opposite side of the room. This layout resulted in constant peripheral activity during experiments. Location 3, a clinic-adjacent lobby with heavy foot traffic, experienced constant background activity as both the public and clinical staff walked around the camera setup. Location 5 was buzzing with activity as hundreds of event attendees explored the space surrounding the MLMC camera setup. For crowd control we used brightly colored cones and strategically positioned equipment to discourage activity too close to the cameras, yet the occasional rogue bystander did persist and walk through the capture volume while data acquisition was in progress. Regardless of this uncontrolled background activity, a full-body 3D kinematic model was created for all study participants.

For MLMC to truly be useful in clinical settings such as rehabilitation gyms, the Theia3D algorithm must also successfully estimate the 3D pose of the studied individual in the presence of additional persons and objects within the motion capture space. Several individuals who met the inclusion criteria of this study had the potential to become unsteady on their feet while completing the walking tasks. In these cases, per experimental protocol, a member of our team acted as a safety spotter trailing approximately arm distance from the participant through the capture volume. In each of these data sets, a 3D biomechanical model was defined for both the studied individual and spotter. Several individuals completed the walking tasks with the use of an assistive device (walker or cane) where there was potential to obstruct the frontal view of the lower body. The MLMC system did produce a full-body 3D kinematic model for these individuals.

The ability to mobilize the MLMC system into local community environments enabled us to study a broader and more diverse sample representing the demographics of the surrounding community. The final data set was comprised of 166 subjects (99 female) aged between 9 and 87 years [mean 43.7, (S.D. 20.4)]. The demographic composition included ethnicity (13.3% Hispanic or Latino, 82.5% Not Hispanic or Latino); race (1.8% American Indian/Alaska Native, 21.1% Asian, 4.8% Black or African American, 0.6% Native Hawaiian or Other Pacific Islander, 59.6% White, 6.0% multiracial); biological sex (59.6% female, 40.4% male); and gender (57.8% Female, 40.4% Male, 1.2% Genderqueer/Gender Non-Conforming). The health history of the sample population included 3 stroke survivors, 3 people with spinal cord injuries, 4 people with vertigo, and 16 people with a history of concussion. Six percent of our sample reported a history of neurologic impairment including, but not limited to: brain lesion, Parkinson’s disease, tremor, or peripheral nerve impairment; 7.8% reported a joint replacement of the hip or knee; and 60.2% indicated a major musculoskeletal injury including, but not limited to: broken bones, torn ligaments, and muscle or tendon injury. Three individuals completed the walking tasks with the use of an assistive device (walker or cane).

Our study focused on the mobilization of the MLMC system by testing it in different locations. However, the feasibility measures of this study suggest this system could be implemented as a highly productive gait assessment tool positioned in busy rehabilitation gyms and clinical spaces on a longer term basis. Cameras could be securely mounted at the edge of the space, and used when needed, regardless of other background activity in the space. Here the utility of the system is demonstrated by the minimal investment required of the participant, who is already at the clinic location for treatment and need only perform the prescribed movement tasks. It is possible to provide outcome measures the day of the test assessment, and also provide prolific periodic assessments for the purposes of longitudinal monitoring.

### Spatiotemporal Parameter Agreement

To aid clinical interpretation, we assessed the agreement of spatiotemporal parameters measured by the MLMC system in comparison to a standard PSW system. Our results showed that, when implemented outside the laboratory, spatiotemporal parameters measured using MLMC showed good to excellent agreement with the PSW system. These findings are consistent with the pressure-sensitive gait mat comparison performed inside the laboratory (Kanko et al., 2021b). Cadence, speed, step length, step time, stride length, and stride time revealed remarkably similar measurements. Even though the length of the walkway used by the PSW and MLMC system was similar (approximately 6 m) the MLMC system detected on average 57.6% more gait cycles for SS task, and 64.9% for FC task. This was because the partial foot falls at the edges of the PSW walkway were excluded from analysis. Increasing the length of the active sensor portion of the PSW system would increase the number of full gait strides. However, the active portion of our walkway was comparable in length to commercially available gait mats used widely in clinical and research facilities.

Similarities in temporal measurements between the MLMC and PSW are notable. However, considering the different sampling frequencies of the two systems (PSW – 100 Hz, MLMC – 60 Hz), future work should assess activities at frequencies higher than walking (i.e., running). There is a consistent asymmetry in all temporal measures whose definitions includes toe off (TO). This is observed in the average mean difference, where stance time is shifted slightly negative and swing time slightly positive (Tables 2A,B). This offset (0.02 s) could be explained by the different sampling frequencies. Of note, stride time does not reflect this offset but does not include TO in its definition. One potential reason for the small offset is the pressure threshold (10 kPa) used by the PSW system to detect foot contact with the floor. The largest mean difference calculated for all temporal metrics was 0.02 s, a value much lower than the 0.07 s for double support time measured by Kanko et al. (2021b) in the lab setting. We report the stance phase of the gait cycle as three distinct epochs: (1) 1^st^ double limb support, (2) single limb support, and (3) 2^nd^ double limb support. Differentiation between the first and second periods of double limb support is an important feature of gait representing limb loading/weight acceptance and pre-swing functions of gait, respectively (Perry and Burnfield, 2010).

Spatial measurements in the direction of walking progression (step length and stride length) were virtually the same between the two systems. The slight difference in stride width between the two systems is not surprising. The foot position defined by the PSW system outlines the entire foot, whereas the MLMC system modeled the foot segment whose proximal position starts at the ankle joint center.

### Case Studies – Detecting Neuropathology With 3D Kinematics

A small, but significant, subset of our community dwelling sample self-reported neuropathologies. Here we examine three case examples: a 61 year old male with Parkinson’s Disease; a 77 year old female stroke survivor with resulting hemiparesis who used a wheeled walker; and a 66 year old male with an incomplete spinal cord injury (iSCI) to the thoracic region. From our full sample, a subset of 28 participants (18 female) aged 9 to 67 years [mean 29.8 (S.D. 13.9)] self-reported no history of neurologic, musculoskeletal, or other health-related conditions. Data from this subset were used for reference comparison.

#### Spatiotemporal Parameters

**FIGURE 2:**
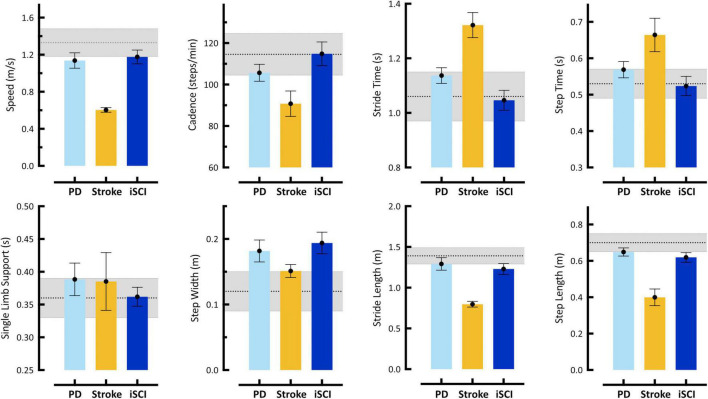
Spatiotemporal gait parameters acquired using markerless motion capture during self-selected (SS) walking task. Bars represent single subject mean (SD) for: Case 1 – 61 year old male with Parkinson’s disease (PD), Case 2 – 77 year old female stroke survivor with hemiparesis (Stroke), and Case 3 – 66 year old male with incomplete Spinal Cord Injury (iSCI). Gray shaded region in each plot represents mean (SD) from our reference control group. Error bars of individual subject means reflect SD of all steps taken. Individual cases are discussed in detail in Section “Case Studies – Detecting Neuropathology with 3D Kinematics.” These data reveal differences with respect to reference controls in one (PD, iSCI) to six (Stroke), but not all eight, spatiotemporal parameters in any individual. Notably, analysis of spatiotemporal variables only might not identify Cases 1 and 3 with neurologic pathologies. Furthermore, these descriptive differences provide little-to-no explanation regarding the causal mechanism of gait dysfunction.

Illustrated in Figure 2 are eight spatiotemporal parameters quantified using MLMC including: speed, cadence, stride time, step time, single limb support time, step width, stride length, and step length. In the first case, the 61 year old gentleman with Parkinson’s Disease (PD), spatiotemporal parameters reveal few differences in comparison to our reference population. Speed, stride length, and step length in this individual reach the low boundary of our reference range, while only step width falls above the reference range. On their own, these spatiotemporal parameters do not provide evidence of significant gait dysfunction in this individual. In the second case, the 77 year old woman with post-stroke hemiparesis, speed, cadence, stride time, step time, stride length, and step length fall outside the reference range, recaptiulating her slower than normal walking speed. Notably, for this individual both step width and single limb support time fall within the reference range, a counterintuitive observation given her underlying pathology, but potentially explained by use of a wheeled walker for external support. For the third case, the 66 year old gentleman with iSCI, spatiotemporal parameters fall within the reference range, with the exception of step width, which is distinctly higher, and stride and step length, both of which reach the lower boundary.

#### 3D Kinematics

**FIGURE 3:**
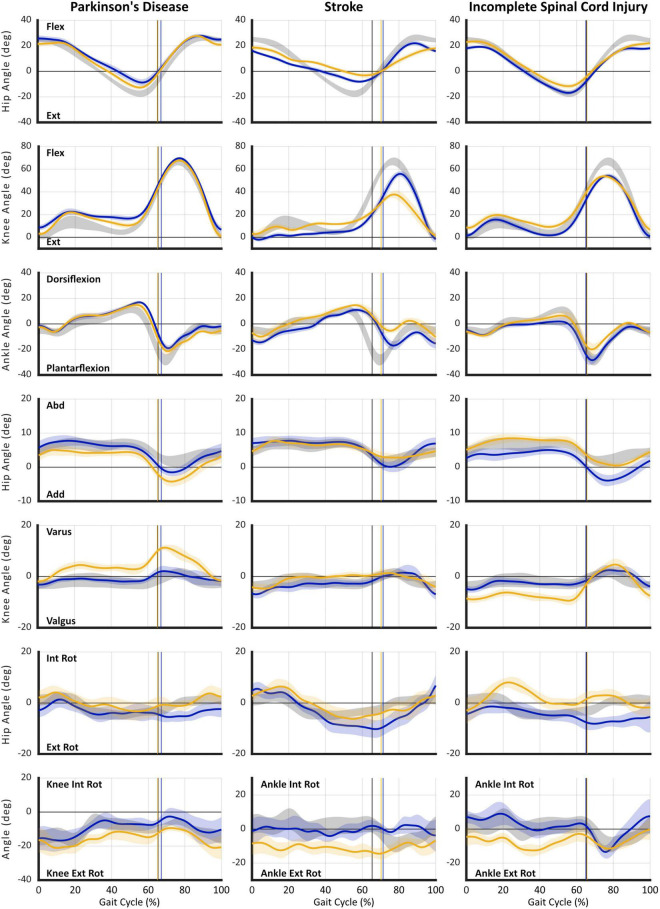
Gait kinematics acquired using markerless motion capture during self-selected (SS) walking task. Plots are presented in columns for: (1) Case 1 – Parkinson’s disease (PD), (2) Case 2 – Stroke, and (3) Case 3 – incomplete Spinal Cord Injury (iSCI). Joint angle curves are presented in rows: (1) hip sagittal plane (flexion/extension), (2) knee sagittal plane (flexion/extension), (3) ankle sagittal plane (dorsiflexion/plantarflexion), (4) hip frontal plane (abduction/adduction), (5) knee frontal plane (varus/valgus), (6) hip transverse plane (internal/external rotation), and (7) transverse plane (internal/external rotation) angles for: knee – Case 1 | PD, or ankle (Cases 2–3) | Stroke and iSCI. For all plots: gray shaded area reflects reference control group ensemble average (SD); gold individual subject right leg; and blue individual subject left leg. Vertical cursor at ∼63% gait cycle denotes toe off for the respective group or leg. Individual subject curves falling outside reference range can be interpreted as gait deviations. Visualization of data across multiple concurrently occurring joint angles/axes enables interpretation of causal mechanisms of gait dysfunction and informs targeted rehabilitation interventions. Furthermore, longitudinal studies enable use of gait kinematics as a sensitive outcome measure specific to the individual’s impairments. All data were acquired using markerless motion capture (Theia3D) with participants wearing their usual attire and footwear.

Illustrated in Figure 3 are hip, knee, and ankle kinematics in sagittal, frontal, and transverse planes acquired with MLMC during SS for the same three cases described above. All data are time-normalized to the gait cycle and presented against reference controls to aid interpretation.

In contrast to expectation established by spatiotemporal parameters, the first case, *Parkinson’s disease*, illustrates a notable asymmetry at all three joints: hip, knee, and ankle. Prominent sagittal plane deviations include: deficient hip extension, distorted knee flexion/extension curve in stance indicative of walking on flexed knees (L > R), delayed and deficient ankle plantarflexion, and failure to attain neutral dorsiflexion in terminal swing (R > L). Frontal plane angles reveal excessive hip circumduction (R > L) and knee varus (R), both of which are known compensatory strategies to effect limb shortening and assure foot clearance during swing. Transverse plane rotations also reveal deviations at the hip (L) and knee (R) as additional compensations for deficient hip and knee extension in stance and reduced foot clearance in swing. Importantly, concurrent evaluation of data from all three joints and planes of motion reveals the mechanisms of critical gait deficiencies. This information could inform development of targeted intervention strategies, improve rehabilitation efficacy, and provide sensitive outcome metrics.

As might be expected, in the second case, *post-stroke hemiparesis*, sagittal plane deviations reveal significant asymmetry and salient gait deviations. Notable, however, are not only deficiencies in the magnitude of joint angles at key gait events, but disrupted timing of the gait pattern, particularly attainment of peak hip flexion near the stance-to-swing transition and delayed plantarflexion. Again, ankle dorsiflexion fails to reach neutral in late/terminal swing bilaterally, although curiously, foot clearance is worse in the less affected (L) side. In this individual, prominent compensations occur in the transverse plane including excessive external hip rotation (L) and ankle rotation (R), both of which are compensations that can contribute to limb shortening.

Spatiotemporal parameters suggest minimal gait impairment for the third case, the individual with *iSCI*. Yet, kinematics reveal obvious deviations in all three planes of motion that fall outside the reference and error ranges. In the sagittal plane, the timing of peak hip flexion is disrupted bilaterally, while the magnitude of hip range of motion is deficient in both flexion (L > R) and extension on the right side. Also on the right side, the timing and magnitude of stance phase knee flexion are aberrant indicating excessive flexion in midstance on the right; both sides reveal markedly reduced peak knee flexion during swing. At the ankle, deficient dorsiflexion in stance indicates failure of tibial advancement. Right sided plantarflexion is delayed and reduced and, again, insufficient dorsiflexion in late swing indicates problematic foot clearance. Combined frontal and transverse plane deviations reflecting exaggerated hip internal rotation, knee valgus, and ankle external rotation (R) during stance place the limb in a guarded and close-packed position signaling compensation for weakness.

#### How Do 3D Kinematics Add Value for Neurorehabilitation?

Taken together, the three cases of individuals affected with neuropathologies illustrate the limitations of relying solely on information provided by spatiotemporal parameters. A variety of tools now available (pressure-sensitive gait mat, mounted inertial measurement sensors, step counter; Hausdorff et al., 1997; Hollman et al., 2011; Fang et al., 2018) make efficient acquisition of spatiotemporal parameters feasible in clinical settings. Yet, it remains unclear how many of these parameters are acquired, interpreted, and used for clinical decision making or outcomes assessment. Even in an ideal setting where such measures are systematically acquired, spatiotemporal parameters merely describe the presence of gait differences without understanding of the underlying cause(s). Moreover, spatiotemporal data are not sufficient to inform development of targeted neurorehabilitation interventions.

To underscore this point, our case examples of Parkinson’s disease and iSCI reveal some spatiotemporal parameters slightly outside of the reference range, but these do not fully illustrate the nature of these individuals’ gait and mobility impairments. Based on spatiotemporal parameters alone, the gentleman with Parkinson’s disease might be prescribed a generalized exercise program to improve his fitness with the goal of increasing walking speed, step and stride length, and conceivably reducing gait variability. In contrast, kinematic analysis suggests multiple targets of musculoskeletal intervention to assure, potentially restore, optimal joint mobility at the hips, knees (L > R), and ankles. While some perspectives might recommend an orthotic to assure foot clearance in terminal swing, an alternative would be further kinematic analysis to understand the dynamic hip-knee interaction at the stance-to-swing transition (Little et al., 2014). Kinematic data available from MLMC make such analysis tractable and so doing would generate a high level of insight regarding effective strategies for successful neurorehabilitation of this individual.

Due to long standing sequelae and multiple co-morbid conditions reported by the stroke survivor presented in our second case, expectations of neurorehabilitation should be realistic. Even so, strategies targeting reduced hip range of motion and deficient ankle plantarflexion on the more involved (right) side would mitigate problems with foot clearance and limb advancement (Little et al., 2014) while concurrently increasing gait speed (Jonkers et al., 2009), stride and step length, and reversing secondary compensations leading to inevitable musculoskeletal deterioration.

Perhaps most noteworthy of these examples is the third case, the gentleman with iSCI, whose modest spatiotemporal deviations might typically be attributed to aging. As a result, any significant concern regarding more severe underlying sensorimotor impairments might be overlooked and left unaddressed by neurorehabilitation. Instead, frontal and transverse plane kinematics reveal a multi-joint compensation most likely due to weakness or more severe pathology on his right side. Multiple, targeted neurorehabilitation strategies could be used to address these impairments. Attention to musculoskeletal impairments is not intended to overshadow the use of MLMC to evaluate interventions that target coordination and timing of motor activity, which may be more familiar approaches for certain neuropathologies. Our purpose with the current study is not to prescribe or promote specific rehabilitation approaches, but rather to consider current and evolving tools and how they can enhance understanding and better inform neurorehabilitation.

The utility of any emerging technology for assessment of gait, mobility, or balance in age-related and neurologic conditions lies in its capacity to provide information regarding detection of underlying disease conditions, disease progression, and clinical outcome. For utility in neurorehabilitation, specifically, it is necessary to acquire information regarding the underlying mechanisms contributing to altered gait and balance parameters. While traditional 3D motion capture methods offer these attributes, the tools, resources, and expertise required for their use have limited its accessibility and feasibility for use in clinical and rehabilitation settings. Here we provide evidence for feasibility of using MLMC in public, participant-facing environments including settings similar to busy, high activity rehabilitation clinics.

In this study, we took MLMC technology and ventured outside the traditional motion capture lab and into the ‘real world’ of our local community to assess characteristics of gait in more ecologically valid environments. These first experiments provide evidence of feasibility including not only its use in community environments, but feasibility of acquiring robust 3D kinematic data from a diverse population. Of note, data presented here show it is now feasible to acquire high resolution 3D motion data in the clinical or clinic-adjacent setting. The technology can be brought *to* the patient/client/participant rather than vice versa, and high-resolution data can be acquired without interruption to regular clinical operations. Access to this information offers unparalleled advantages for the rehabilitation clinician managing cases of complex neuromotor dysfunction. Rather than relying on observation, gross approximations (e.g., clinical scales), estimates (e.g., gait speed, spatiotemporal parameters), or proxy measures of clinical status to understand motor dysfunction, the high-resolution information generated from 3D motion capture makes it possible to develop rehabilitation strategies based on the individual’s specific movement deviations. Whether neurorehabilitation targets strengthening strategies, pharmacologic agents, or biologic interventions such as stem cell infusions, 3D gait analysis provides a robust and sensitive digital biomarker. This foundational work enables future investigation with MLMC especially its use as a digital biomarker of disease progression and rehabilitation outcome.

## Data Availability Statement

The raw data supporting the conclusions of this article will be made available by the authors, without undue reservation.

## Ethics Statement

The studies involving human participants were reviewed and approved by University of California, Davis Institutional Review Board. Written informed consent to participate in this study was provided by the participants’ legal guardian/next of kin.

## Author Contributions

CP and TM conceived and designed the study, wrote the original manuscript, and interpreted data. TM and WS processed data. TM analyzed the data. All authors contributed to experimental implementation and data collection, reviewed, and approved the final manuscript.

## Conflict of Interest

The authors declare that the research was conducted in the absence of any commercial or financial relationships that could be construed as a potential conflict of interest.

## Publisher’s Note

All claims expressed in this article are solely those of the authors and do not necessarily represent those of their affiliated organizations, or those of the publisher, the editors and the reviewers. Any product that may be evaluated in this article, or claim that may be made by its manufacturer, is not guaranteed or endorsed by the publisher.
